# Isolation and characterization of the first phage infecting ecologically important marine bacteria *Erythrobacter*

**DOI:** 10.1186/s12985-017-0773-x

**Published:** 2017-06-07

**Authors:** Longfei Lu, Lanlan Cai, Nianzhi Jiao, Rui Zhang

**Affiliations:** State Key Laboratory of Marine Environmental Science, Institute of Marine Microbes and Ecospheres, Xiamen University (Xiang’an), Xiamen, Fujian 361102 China

**Keywords:** *Erythrobacter*, Marine, Siphovirus, Complete genome sequence

## Abstract

**Background:**

*Erythrobacter* comprises a widespread and ecologically significant genus of marine bacteria. However, no phage infecting *Erythrobacter* spp. has been reported to date. This study describes the isolation and characterization of phage vB_EliS-R6L from *Erythrobacter*.

**Methods:**

Standard virus enrichment and double-layer agar methods were used to isolate and characterize the phage. Morphology was observed by transmission electron microscopy, and a one-step growth curve assay was performed. The phage genome was sequenced using the Illumina Miseq platform and annotated using standard bioinformatics tools. Phylogenetic analyses were performed based on the deduced amino acid sequences of terminase, endolysin, portal protein, and major capsid protein, and genome recruitment analysis was conducted using Jiulong River Estuary Virome, Pacific Ocean Virome and Global Ocean Survey databases.

**Results:**

A novel phage, vB_EliS-R6L, from coastal waters of Xiamen, China, was isolated and found to infect the marine bacterium *Erythrobacter litoralis* DSM 8509. Morphological observation and genome analysis revealed that phage vB_EliS-R6L is a siphovirus with a 65.7-kb genome that encodes 108 putative gene products. The phage exhibits growth at a wide range of temperature and pH conditions. Genes encoding five methylase-related proteins were found in the genome, and recognition site predictions suggested its resistance to restriction-modification host systems. Genomic comparisons and phylogenetic analyses indicate that phage vB_EliS-R6L is distinct from other known phages. Metagenomic recruitment analysis revealed that vB_EliS-R6L-like phages are widespread in marine environments, with likely distribution in coastal waters.

**Conclusions:**

Isolation of the first *Erythrobacter* phage (vB_EliS-R6L) will contribute to our understanding of host-phage interactions, the ecology of marine *Erythrobacter* and viral metagenome annotation efforts.

## Background

As ecologically significant marine bacteria, *Erythrobacter* species (Alphaproteobacteria) are frequently detected in and isolated from nutrient-rich coastal seawaters [1–5]. Moreover, these microorganisms are thought to comprise a major fraction of the marine photoheterotrophs known as aerobic anoxygenic phototrophic bacteria (AAPBs), which play a significant role in the cycling of both organic and inorganic carbon in the ocean [2, 6–8]. To date, 19 *Erythrobacter* species have been reported, and genomic and metabolic studies have shown that members of this genus are metabolically versatile [5, 9–11]. The first marine *Erythrobacter* isolate was *E. longus* DSM 6997, which was also the first AAPB identified [1]. In 1994, *E. litoralis* DSM 8509, containing the carotenoids bacteriorubixanthinal and erythroxanthin sulfate, was isolated from a marine cyanobacterial mat [12]. In addition, previous studies have demonstrated the potential use of *Erythrobacte*r species (e.g., *E. longus* and *E. citreus*) for bioremediation of alkane contamination [13]. These species show high levels of resistance to tellurite and accumulate metallic tellurium crystals (e.g., *E. longus*) [14]; enantioselective epoxide hydrolase activity (e.g., *E. longus*) has also been reported [15].

Bacteriophages (viruses that infect bacteria) have important roles in the abundance, activity, and diversity of bacterial communities [16–18], and isolation and genomic characterization of phages greatly improves our understanding of the ecology and evolution of their hosts. For example, cyanophages (viruses that infect cyanobacteria) are active and abundant agents of mortality that directly affect the distribution and species composition of cyanobacteria in the aquatic environment [17, 19]. In addition, investigation of SAR11 viruses helped to show that the highly abundant distribution of these viruses is the result of adaptation to resource competition [20]. It has also been suggested that roseophages (viruses that infect *Roseobacter* species, another representative genus of Alphaproteobacteria) can quickly alter the growth and abundance of their host population by changing their infection strategy and can shunt bacterial secondary production into the environmental dissolved-carbon pool [e.g., [21, 22].

Isolation of novel phages can assist with both the annotation of unidentified functional genes and in the discovery of diverse and widespread viral assemblages in aquatic and marine environments through virome database query [20, 22, 23]. However, no phage infecting *Erythrobacter* has been reported to date, hindering an integrated understanding of the life cycle of these microbes in the ocean. In this study, we report the first isolation of a novel phage infecting *E. litoralis* DSM 8509.

## Methods

### Bacterial strains and growth conditions

All of the bacterial strains used in this study are listed in Table 1. *E. litoralis* DSM 8509 and other strains were cultivated at 30 °C in RO medium, an artificial seawater medium containing 1 g/L yeast extract, 1 g/L tryptone, and 1 g/L sodium acetate at pH 7.5 [24].

**Table 1:** Bacterial strains used in the host-range test and their susceptibility to the phage vB_EliS-R6L

Strains	Best matched species (% Id of 16S rDNA)	Source and location	References	Susceptibility to phage vB_EliS-R6L	Efficiency of plaquing
*Erythrobacter litoralis* DSM 8509*		Cyanobacterial mat, Netherlands	[12]	+	100%
*Erythrobacter longus* DSM 6997*		Seaweed *Enteromorpha linza*, Japan	[1]	+	94.74 ± 3.78%
*Erythrobacter* sp. JL 475		Surface sea water, South China sea, China	[11]	-	-
JL 2316	*Erythrobacter* sp. CC-AMZ-30 L (97.12)	Surface sea water, Pacific Ocean		-	-
JL 967	*Erythrobacter* sp. M71_W20 (100.00)	Surface sea water, Taiwan strait, China		-	-
JL 1267	*Erythrobacter* sp. MON004 (100.00)	Surface sea water, South China sea, China		-	-
JL 971–1	*Erythrobacter nanhaisediminis* (99.33)	Surface sea water, Taiwan strait, China		-	-
JL 1059	*Erythrobacter nanhaisediminis* T30 (99.22)	Upper sea water (150 m), West Pacific Ocean		-	-
JL 1033	*Erythrobacter nanhaisediminis* T30 (99.69)	Upper sea water (50 m), West Pacific Ocean		-	-
JL 1302	*Erythrobacter nanhaisediminis* T30 (97.79)	Surface sea water, South China sea, China		-	-
JL 1201	*Erythrobacter vulgaris* TVG01-C004 (99.80)	Surface sea water, West Pacific Ocean		-	-
JL 274–1	*Erythrobacter vulgaris* 022 2–10 (99.22)	Changjiang Estuary, China		-	-
JL 1500	*Erythrobacter pelagi* UST081027–248 (99.90)	Surface sea water, Beibu Gulf, China		-	-
JL 1463	*Erythrobacter pelagi* UST081027–248 (98.48)	Surface sea water, South China sea, China		-	-
JL 883	*Erythrobacter flavus* SW-46 (99.79)	Surface sea water, Taiwan strait, China		-	-
JL 923	*Erythrobacter flavus* SW-46 (99.25)	Surface sea water, South China sea, China		-	-
JL 1833	*Erythrobacter flavus* BL16 (100.00)	Bottom sea water, South China sea, China		-	-
JL 1408	*Erythrobacter flavus* SW-46 (99.89)	Surface sea water, South China sea, China		-	-
JL 917	*Erythrobacter citreus* RE35F/1 (99.72)	Surface sea water, Taiwan strait, China		-	-
JL 1317	*Erythrobacter flavus SW-46* (99.01)	Surface sea water, South China sea, China		-	-
JL 658–2	*Erythrobacter citreus* RE35F/1 (99.66)	Surface sea water, Taiwan strait, China		-	-
*Roseobacter denitrificans* OCh114 DSM 7001*		Seaweed, Japan	[52]	-	-
*Dinoroseobacter shibae* DFL12*		Cells of *Prorocentrum lima*	[53]	-	-
*Citromicrobium bathyomarinum* JL 354		Surface sea water, South China sea, China		-	-
JL 1363	*Citromicrobium* sp. (100.00)	Upper sea water (50 m), South China sea, China		-	-
JL 2210	*Lutibacterium* sp. (100.00)	Surface sea water, Atlantic Ocean		-	-
JL 1614	*Halomonas* sp. (100.00)	Surface sea water, Pacific Ocean		-	-

*strains were purchased from DSMZ (the German Resource Center for Biological Material), Germany. +, cell lysis; −, no effect

### Isolation of the phage

Phage vB_EliS-R6L was isolated from seawater obtained in March 2014 off the coast of Xiamen, China (118°04′ E, 24°31′ N), using standard virus enrichment and double-layer agar methods. Briefly, *E. litoralis* DSM 8509 (100 mL) was co-cultured with a pre-filtered (0.22-μm membrane filter; Millipore, USA) seawater sample (20 mL) for 24 h at 30 °C. The culture was filtered again and serially diluted to determine phage activity using a double-layer agar method [25]. A single plaque was collected from the plate using a sterile pipette (Fisher, Canada) and then purified four successive times using the double-layer agar method. Following purification, stock cultures of the phage were prepared using sodium chloride-magnesium sulfate (SM) buffer (100 mM NaCl, 50 mM Tris, 10 mM MgSO_4_, and 0.01% gelatin, pH 7.5) supplemented with several drops of chloroform and stored at 4 °C and −80 °C.

### Transmission electron microscopy (TEM)

For TEM analysis, 1 L of *E. litoralis* DSM 8509 culture (OD600 = 0.5) was inoculated with the phage at a multiplicity of infection of 10 and cultivated for 24 h at 30 °C. The mixture was centrifuged at 6000×*g* for 10 min, and the upper aqueous phase was filtered through a 0.22-μm membrane and precipitated with 10% (*w*/*v*) dissolved polyethylene glycol 8000 (containing 1 M NaCl). After >8 h at 4 °C, the mixture was centrifuged at 10,000×*g* for 50 min at 4 °C, and the pellet was gently resuspended in 5 mL of SM buffer. The phages were then purified by CsCl gradient ultra-centrifugation (gradient-density: 1.5 g/mL, 200,000×*g*, 24 h, 4 °C; Optima L-100 XP Ultracentrifuge, Beckman Coulter). The purified phage particles were collected and dialyzed twice in SM buffer; 20 μL of suspension was added dropwise onto a copper grid and negatively stained with 2% aqueous uranyl acetate for 10 min. Transmission electron micrographs were obtained using a JEM-2100HC transmission electron microscope (JEOL, Japan) at an accelerating voltage of 120 kV. The phage size was calculated from at least 20 particles.

### Chloroform sensitivity

To determine whether phage vB_EliS-R6L contains lipids, its sensitivity to chloroform was examined as described previously [26]. Briefly, 500 μL of the phage suspension (~10^9^ plaque forming units (PFU)/mL) were mixed with 5 μL, 50 μL, or 500 μL of chloroform, vigorously shaken for 2 min, and then incubated at 30 °C for 30 min. The samples were immediately diluted and plated for phage titration using double-layer agar plates inoculated with *E. litoralis* DSM 8509.

### Host range analysis

To investigate the host range of phage vB_EliS-R6L, plaque assays were performed on 27 marine bacterial strains, including 21 *Erythrobacter* strains, two *Citromicrobium* strains, and one each of the genera *Roseobacter*, *Dinoroseobacter*, *Lutibacterium*, and *Halomonas* (Table 1). The host range was determined by adding 5 μL of a diluted phage suspension (~10^7^ PFU/mL) dropwise onto the surface of double-layer agar plates inoculated with the bacterial strain of interest. The plates were incubated at 30 °C for up to 7 days, and plaque formation was assessed repeatedly during this period. The efficiency of plating (EOP) of susceptible strains was quantified by calculating the ratio of the PFU obtained with each phage-susceptible strain to the PFU obtained with *E. litoralis* DSM 8509. All assays were carried out in triplicate.

### One-step growth assays

One-step growth curve experiments were performed as previously described [25, 27]. Briefly, mid-exponential phase *E. litoralis* DSM 8509 (optical density at 600 nm = 0.3–0.5, 100 mL) was inoculated with phage at a multiplicity of infection of 0.01 and allowed to adsorb for 10 min at 30 °C. The mixture was then centrifuged at 6000×*g* for 10 min to remove non-absorbed phage in the supernatant; the pelleted cells were resuspended in 100 mL of RO medium, followed by incubation at 30 °C. Two sets of duplicate samples were removed at 20-min intervals for 6 h, and chloroform (1% final concentration) was added to the second set to release the intracellular phage. The two samples were then diluted and immediately plated for phage titration using the double-layer agar plate method. Another set of cultures without phage inoculation served as the blank control. Samples for optical density (OD_600_) measurements from both the treated and untreated cultures were removed at the 20-min intervals for 6 h and at 1-h intervals for the next 4 h. The PFU of each sample was calculated by counting the plaques on the bacterial lawn. The assay was performed in triplicate.

### Thermal/pH stability

To investigate the thermal stability of the phage, 1 mL of phage vB_EliS-R6L (~10 ^7^ PFU/mL) with SM buffer was incubated for 2 h at 30 °C, 40 °C, 42.5 °C, 45 °C, 50 °C, 60 °C, 70 °C, 75 °C, or 80 °C, after which the phage suspensions were immediately cooled to 4 °C for activity estimation. To evaluate the stability of the phage at different pH levels, RO medium was adjusted to pH 1–14 with sterile 5 M HCl or NaOH solution and then filtered through a 0.22-μm membrane filter (Millipore, USA). Additionally, 1 mL of a phage suspension (~10 ^7^ PFU/mL) prefiltered through a 0.22-μm membrane filter was incubated at 30 °C for 24 h in 9 mL RO medium of different pHs. Phage activity was determined using the double-layer agar method with RO medium (pH 7.5) at 30 °C and assessed by calculating changes in PFU following exposure to the different temperatures and pH levels. All assays were performed in triplicate.

### Effects of temperature and pH on infection

To investigate the effect of temperature on phage infection, 5 μL of a phage suspension (~10 ^9^ PFU/mL) was added dropwise onto double-layer agar plates containing *E. litoralis* DSM 8509 and incubated at 15 °C, 20 °C, 25 °C, 27.5 °C, 30 °C, 35 °C, and 40 °C for 7 days. To investigate the effect of pH on infection, the pH of RO medium was adjusted with 5 M HCl (pH 4–5), 0.2 M Na_2_HPO_4_/ NaH_2_PO_4_ (pH 6–8) or 0.1 M NaHCO_3_/Na_2_CO_3_ (pH 9–11); after autoclaving, the pH was checked with pH test paper and readjusted if necessary. Next, 5 μL of a phage suspension (~10 ^9^ PFU/mL) was added dropwise onto double-layer agar plates inoculated with host cells at different pH values. The plates were incubated at 30 °C for up to 7 days. All assays were performed in triplicate.

### Lysogenic/lytic assays

To investigate whether the phage can integrate onto the genome of its host, 10 μL of a phage suspension (~10 ^9^ PFU/mL) was added dropwise onto double-layer agar plates inoculated with *E. litoralis* DSM 8509; the center portion within the plaques was carefully pipetted out and inoculated onto a new plate. After two rounds of isolation and purification, 40 randomly selected bacterial colonies were chosen for colony polymerase chain reaction (PCR) using two pairs of primers, designed according to phage genome annotation, targeting ORF 91 (Major capsid protein) (forward primer 5′ –GCTGACCACCAAGCAGATGA - 3′, reverse primer 5′ - CGGAACGAGGCTATCCCAC - 3′, 521 bp) and ORF 100 (Terminase) (forward primer 5′ - TCATGTGGCAGGCTTGGG - 3′, reverse primer 5′ - GGGTCGGTCCAGTCTTTCG - 3′, 549 bp).

### Phage DNA extraction, sequencing, and genomic analysis

Using the same sample preparation utilized for TEM analysis, 1 mL of a phage suspension was purified by CsCl density-gradient centrifugation, followed by dialysis. To remove free DNA and RNA, the sample was then digested at 37 °C for 1 h with DNase I and RNase A (Takara) at final concentrations of 1 μg/mL. The solution was incubated with proteinase K and sodium dodecyl sulfate at final concentrations of 100 μg/mL and 1% (*w*/*v*), respectively, at 55 °C for 2 h. After incubation, the solution was extracted twice with phenol:chloroform:isoamyl alcohol (25:24:1) and once with chloroform:isoamyl alcohol (24:1), after which the solutions were precipitated with sodium acetate and precooled ethanol at final concentrations of 1/10 and 1/1 (*v*/v), respectively. After overnight incubation at −20 °C, DNA was collected by centrifugation and successively washed twice with precooled 70% and 100% ethanol. The genomic DNA of vB_EliS-R6L was sequenced using the Illumina Miseq platform to generate 2 × 251 bp paired-end reads. The reads were assembled using CLC Genomics Workbench software (18,777 × coverage).

### Genomic and bioinformatic analyses

The GeneMarkS online server (http://exon.gatech.edu/Genemark/genemarks.cgi), Glimmer 3.0 (http://ccb.jhu.edu/software/glimmer/index.shtml), and the ORF Finder online server (https://www.ncbi.nlm.nih.gov/orffinder/) were used to identify putative open reading frames (ORFs). Genes were annotated using BLAST searches against the NCBI non-redundant (nr) protein database, with a cut-off of E-value ≤10^−5^. A temperate and/or lytic lifestyle was predicted using the phage classification toolset (PHACTS) online prediction program (http://www.phantome.org/PHACTS/index.php). Methylase ORFs were searched using the REBASE^R^ online program (http://rebase.neb.com/rebase/rebase.html).

The amino acid sequences of endolysin protein (ORF 74), major capsid protein (ORF 91), portal protein (ORF 98), and terminase (ORF 100) from phage vB_EliS-R6L were used to construct neighbor-joining phylogenetic trees with MEGA 6.06 and 200 bootstrap replications. For use in the phylogenetic analysis, the amino acid sequences of these four proteins from closely related phages were retrieved from GenBank.

### Genome recruitment

To explore the geographic distribution of vB_EliS-R6L-like phages, the amino acid sequences of the phage ORFs were employed as queries to search against metagenomic databases of the Jiulong River Estuary (JRE), Xiamen, China [28], the Pacific Ocean Virome (POV) and Global Ocean Survey (GOS) (http://data.imicrobe.us/) using tBLASTn at a cut-off of E-value ≤10^−5^, an alignment value ≥30 and a score value ≥40. The count abundance of each read was normalized by dividing by the number of total reads in the database and the size of the gene product [20].

### Nucleotide sequence accession number

The genome sequence of phage vB_EliS-R6L was deposited in the GenBank database under accession number KY006853.

## Results and discussion

### Phage isolation and basic characterization

**Fig. 1:**
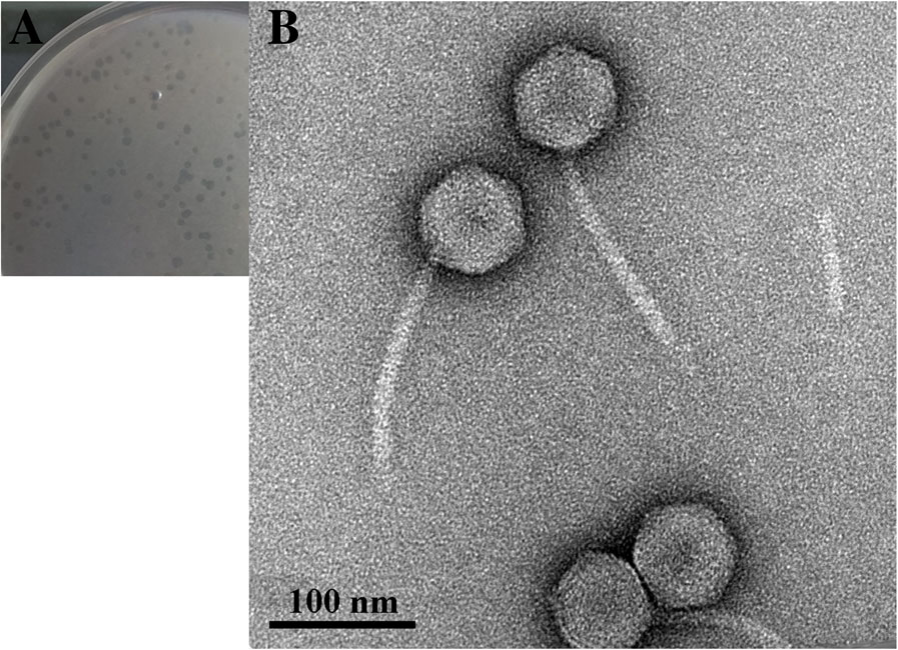
Plaque (**a**) and TEM (**b**) images of *Erythrobacter litoralis* DSM 8509 phage vB_EliS-R6L particles. The scale bar (**b**) equals 100 nm

To the best of our knowledge, vB_EliS-R6L is the first phage isolated from the ecologically important marine bacteria of the genus *Erythrobacter*. vB_EliS-R6L forms small, clear, round (1–4 mm diameter) plaques on a bacterial lawn (Fig. 1). After treatment with different concentrations of chloroform (i.e., 1%, 10%, and 100% (*v*/v)), the phage showed survival rates of 94.7 ± 4.9%, 83.1 ± 2.5%, and 81.9 ± 1.9%, respectively, indicating that vB_EliS-R6L may not sensitive to chloroform or contain lipids. TEM micrographs revealed that it belongs to the siphovirus family, with an icosahedral capsid 75.9 ± 2.2 nm in diameter and a characteristically long tail of 165.6 ± 2.3 nm (Fig. 1).

Of all the strains tested, phage vB_EliS-R6L could only infect *E. litoralis* DSM 8509 and *E. longus* DSM 6997, the only strains for each species that could be obtained from public culture collections. Of the commonly isolated three-tailed phage families (*Myoviridae*, *Siphoviridae,* and *Podoviridae*), *Myoviridae* phages have a broader host range than species of the other two families. Therefore, it was not unexpected that a narrow host range was observed for phage vB_EliS-R6L. Based on whole-genome comparison, Zheng et al. (2016) reported that *Erythrobacter* strains cluster into three groups, with strains DSM 8509, DSM 6997, and JL 475 belonging to the same group. These three strains share high 16S rRNA gene identity (> 97%) but can be discriminated by average nucleotide identity analysis [11]. Integrative and conjugative element analysis showed that DSM 8509 and DSM 6997 cluster closely together and away from JL 475, suggesting asynchronous evolution. This may account for the ability of phage vB_EliS-R6L to infect DSM 8509 and 6997 but not JL 475. In addition, previous studies have suggested that the number of tRNAs can be positively correlated with host range due to compensation for different codon usage patterns in host bacteria [29]. No tRNAs were identified in the phage vB_EliS-R6L genome using tRNAscan-SE (1.3.1) software [30], which may also account for its relatively narrow host range. In lysogenic/lytic assays, 17.5% (7/40) of bacterial isolates from the center portion of plaques showed positive PCR amplification using primers specific for phage ORFs. This finding suggests that vB_EliS-R6L may integrate into its host cell and possibly enter into a lysogenic life cycle, which is consistent with our bioinformatic analysis (see below).

**Fig. 2:**
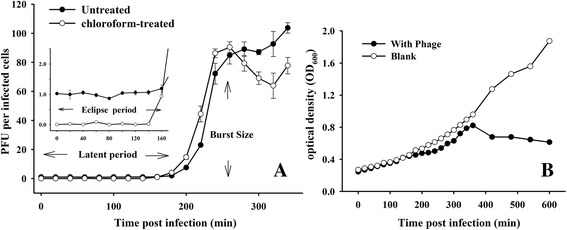
One-step growth curve analysis of *Erythrobacter litoralis* phage vB_EliS-R6L. (**a**) the Plaque forming Unints (PFUs) of the phage and (**b**) the optical density (OD_600_) of *Erythrobacter litoralis* DSM 8509. Open circles (**a**), chloroform-treated samples; closed circles (**a**), non-chloroform-treated samples. Open circles (**b**), without phage-inoculated samples; closed circles (**b**), phage-inoculated samples. OD, optical density

**Fig. 3:**
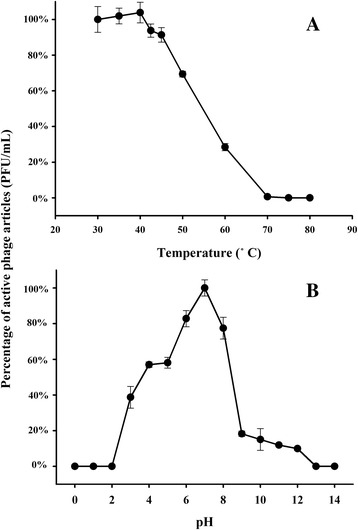
Stability of *Erythrobacter litoralis* phage vB_EliS-R6L under various stress conditions. (**a**) pH stability and (**b**) temperature stability. PFU, Plaque Forming Unit. Error bars show standard deviations among triplicate samples

According to one-step growth curve experiments, the eclipse and latent periods of phage infection occurred at 2 h 40 min and 3 h post-infection, respectively (Fig. 2). The burst size was ~86 PFU/cell, similar to the latent period and burst size of most phages infecting *Roseobacter* species, ranging from <1–6 h and 27–1500 PFU/cell, respectively [23, 31–33]. Stability assessment showed that over 60% of vB_EliS-R6L phage remained active at temperatures up to 50 °C (2 h treatment) and that <1% remained active at temperatures >70 °C (Fig. 3). In addition, the phage survival rate was greater than 77% after 24 h at pH 6, 7, or 8 (Fig. 3). Although vB_EliS-R6L retained some activity after 24 h at pH 3 (39%) and pH 12 (10%), activity was lost below pH 2 or above pH 13. An infection condition test showed that phage vB_EliS-R6L could infect *E. litoralis* DSM 8509 and form clear plaques on plates within 2 days at 25 °C ~ 35 °C. Visible plaques appeared on plates after 4 days at 15 °C and 20 °C, whereas no clear plaques were visible at 40 °C after 7 days of incubation. In addition, plaques were observed in the infection test within a pH range of 7–10. These data showed phage vB_EliS-R6L particles to be stable, with broad temperature and pH tolerance compared to most isolated phages [34], characteristics that might offer more survival opportunities in the diverse marine environment. However, phage vB_EliS-R6L was only able to successfully proliferate within a relatively narrow range of conditions (i.e., < 40 °C, pH 7–10). Unsuccessful infection might be a consequence of thermal/chemical alterations to the phage structure or host receptors [35, 36], and further investigation is needed.

### Genomic analysis of phage vB_EliS-R6L

The complete dsDNA genome of phage vB_EliS-R6L is 65,675 bp in size (GenBank accession no. KY006853). The overall G + C content is 66.5%, similar to that of its host (i.e., 65.2%, GenBank accession no. NZ_CP017057). A total of 108 ORFs were identified (Table 2), and identity of the predicted coding sequences with sequences available in GenBank is low (26–77% at the amino acid level). Homologous sequences in the NCBI non-redundant protein database were found for 58 gene products; however, only 29 had predicted functions (Table 2), 19 of which have been assigned to known functional domain categories. In total, 27 ORFs are homologous with previously identified bacteriophage genes, and 15 are homologs of proteins from siphophage-infecting Alphaproteobacteria. Overall, as suggested by the low degree of coverage (< 3%) of the entire genome sequence identified by BLASTn analysis, the vB_EliS-R6L genome is largely unique compared with other published phage genomes.

**Table 2:** *Erythrobacter litoralis* DSM 8509 phage vB_EliS-R6L genome annotations (KY006853)

Gene	Strand	Start (bp)	Stop (bp)	Residue length (nn)	Residue length (aa)	Putative function/feature	Best matched evidence or organism	Homolog Accession Num.	% Id	BlastP E-Value
1	-	<3	2423	2421	830	DNA modification methylase	uncultured Mediterranean phage uvMED	BAQ92410	55	2.00E-47
2	-	2420	2875	456	151	hypothetical protein (DNA polymerase III beta clamp)	*Methylobacterium* sp. ARG-1 (*Caulobacter* phage Sansa)	WP_050734237 (AKU43506)	49 (27)	7.00E-11 (3.00E-04)
3	-	2883	3239	357	118	hypothetical protein	None	n/a	n/a	n/a
4	-	3286	3456	171	56	hypothetical protein	None	n/a	n/a	n/a
5	-	3453	3614	162	53	hypothetical protein	None	n/a	n/a	n/a
6	-	3601	3813	213	70	hypothetical protein	None	n/a	n/a	n/a
7	-	3810	4853	1044	347	phosphoadenosine phosphosulfate reductase	*Sphingomonas* sp. LH128	WP_008827527	49	3.00E-85
8	-	4835	5023	189	62	hypothetical protein	None	n/a	n/a	n/a
9	-	5020	5628	609	202	molecular chaperone	*Caulobacter* phage Sansa	AKU43478	41	5.00E-42
10	-	5674	6315	642	213	hypothetical protein	*Pseudomonas oryzihabitans*	WP_044342705	55	2.00E-63
11	-	6322	6558	237	78	hypothetical protein	None	n/a	n/a	n/a
12	-	6537	6791	255	84	hypothetical protein	*Sphingomonas* sp. Ant H11	WP_052192475	52	3.00E-23
13	-	6791	7174	384	127	hypothetical protein	uncultured Mediterranean phage uvMED (*Caulobacter* phage Sansa)	BAR28076 (AKU43468)	39 (27)	5.00E-08 (7.00E-04)
14	-	7171	7473	303	100	hypothetical protein	None	n/a	n/a	n/a
15	-	7473	7904	432	143	hypothetical protein	None	n/a	n/a	n/a
16	-	7901	8050	150	49	hypothetical protein	None	n/a	n/a	n/a
17	-	8047	8397	351	116	hypothetical protein	None	n/a	n/a	n/a
18	-	8394	8933	540	179	hypothetical protein	None	n/a	n/a	n/a
19	-	8933	9061	129	42	hypothetical protein	None	n/a	n/a	n/a
20	-	9061	9198	138	45	hypothetical protein	None	n/a	n/a	n/a
21	-	9195	9422	228	75	hypothetical protein	None	n/a	n/a	n/a
22	-	9419	9679	261	86	hypothetical protein P106B_62	*Rhizobium* phage vB_RglS_P106B	YP_009005988	47	2.00E-08
23	-	9733	10,005	273	90	hypothetical protein	None	n/a	n/a	n/a
24	-	10,005	10,118	114	37	hypothetical protein	None (*Caulobacter* phage Sansa)	n/a (AKU43430)	n/a (27)	n/a (2.00E-06)
25	-	10,115	10,603	489	162	hypothetical protein	None	n/a	n/a	n/a
26	-	10,596	11,546	951	316	hypothetical protein	*Rhizobium tropici*	WP_052227599	45	1.10E-02
27	-	11,697	12,263	567	188	hypothetical protein	*Sphingomonas* sp. Y57	WP_053000396	41	3.00E-31
28	-	12,278	12,880	603	200	hypothetical protein	*Novosphingobium* sp. ST904	WP_054436273	39	3.00E-23
29	-	12,867	13,688	822	273	hypothetical protein	*Erythrobacter* sp. SG61-1 L	WP_054529722	40	3.00E-10
30	-	13,961	14,176	216	71	hypothetical protein	None	n/a	n/a	n/a
31	-	14,173	14,859	687	228	hypothetical protein	*Lactobacillus* phage LL-H	YP_001285924	43	1.00E-18
32	-	14,856	15,137	282	93	acyl carrier protein	*Ruminococcus albus*	WP_037276568	44	6.00E-13
33	-	15,195	15,818	624	207	hypothetical protein	*Sphingomonas wittichii*	WP_016745765	29	6.00E-10
34	-	15,808	16,488	681	226	methyltransferase	*Caulobacter* phage Sansa	AKU43482	31	2.00E-08
35	-	16,488	16,703	216	71	hypothetical protein	None	n/a	n/a	n/a
36	+	16,834	17,106	273	90	hypothetical protein	None	n/a	n/a	n/a
37	+	17,106	17,384	279	92	hypothetical protein	None	n/a	n/a	n/a
38	+	17,381	17,662	282	93	hypothetical protein	None	n/a	n/a	n/a
39	+	17,664	17,909	246	81	hypothetical protein	None	n/a	n/a	n/a
40	+	17,986	18,774	789	262	type I restriction-modification system methyltransferase subunit-like protein	*Methylobacterium nodulans* ORS 2060	YP_009126070	41	2.71E-44
41	+	18,774	20,180	1407	468	nucleoside triphosphate hydrolase	*Caulobacter* phage Sansa	AKU43472	37	1.00E-59
42	+	20,177	20,440	264	87	hypothetical protein	*Sphingomonas* sp. BHC-A	WP_025772726	51	1.00E-13
43	+	20,437	20,730	294	97	hypothetical protein	None	n/a	n/a	n/a
44	+	20,723	20,983	261	86	hypothetical protein	None	n/a	n/a	n/a
45	+	20,980	22,629	1650	549	nucleic acid-binding protein	*Caulobacter* phage Sansa	AKU43470	30	2.00E-12
46	+	22,626	23,351	726	241	exonuclease	*Sphingobium baderi* LL03 (*Caulobacter* phage Sansa)	KMS62764 (AKU43467)	46 (35)	8.00E-64 (1.00E-22)
47	+	23,341	24,513	1173	390	ERF family protein	*Dunaliella viridis* virus SI2	YP_009021005	31	1.00E-19
48	+	24,513	24,677	165	54	hypothetical protein	None	n/a	n/a	n/a
49	+	24,677	25,180	504	167	single-stranded DNA-binding protein	*Citromicrobium* (*Caulobacter* phage Sansa)	WP_010236565 (AKU43479)	63 (52)	2.00E-56 (1.00E-52)
50	+	25,192	26,049	858	285	phage Gp37Gp68 (ssDNA-annealing protein)	*Sphingomonas* sp. Y57 (*Caulobacter* phage Sansa)	WP_047169428 (AKU43469)	52 (30)	3.00E-89 (9.00E-05)
51	+	26,046	26,573	528	175	hypothetical protein	None	n/a	n/a	n/a
52	+	26,598	27,239	642	213	hypothetical protein	None (*Caulobacter* phage Sansa)	n/a (AKU43520)	n/a (23)	n/a (3.00E-04)
53	+	27,232	27,639	408	135	hypothetical protein	*Sphingobium chungbukense*	WP_046763480	43	7.00E-15
54	-	27,873	28,238	366	121	hypothetical protein	*Pseudomonas aeruginosa*	WP_052157666	48	2.00E-11
55	+	28,309	28,692	384	127	cytosine-specific methyltransferase	*Ralstonia solanacearum* GMI1000	NP_518991	48	7.98E-23
56	+	28,730	28,918	189	62	hypothetical protein	None	n/a	n/a	n/a
57	+	28,918	29,508	591	196	hypothetical protein	None	n/a	n/a	n/a
58	+	29,612	30,007	396	131	hypothetical protein	None	n/a	n/a	n/a
59	+	30,092	30,574	483	160	hypothetical protein	None	n/a	n/a	n/a
60	+	30,574	30,828	255	84	hypothetical protein	None	n/a	n/a	n/a
61	+	30,903	31,304	402	133	MucR family transcriptional regulator	*Methylobacterium nodulans*	WP_012631401	55	3.00E-33
62	+	31,301	31,687	387	128	hypothetical protein	None	n/a	n/a	n/a
63	+	31,684	31,902	219	72	hypothetical protein	None	n/a	n/a	n/a
64	+	31,893	32,033	141	46	hypothetical protein	None	n/a	n/a	n/a
65	+	32,020	32,235	216	71	hypothetical protein	None	n/a	n/a	n/a
66	+	32,235	32,468	234	77	hypothetical protein	None	n/a	n/a	n/a
67	+	32,465	33,223	759	252	DNA methylase	*Mycobacterium* phage Llama	AIM51011	56	2.00E-51
68	+	33,259	33,612	354	117	hypothetical protein	*Vibrio* phage VvAW1	YP_007518376	44	2.00E-20
69	+	33,662	33,958	297	98	hypothetical protein	*Burkholderia vietnamiensis*	WP_011875349	34	1.00E-05
70	+	33,955	34,455	501	166	hypothetical protein	*Novosphingobium* sp. KN65.2	CDO34010	41	4.00E-22
71	-	34,717	34,962	246	81	hypothetical protein	*Sphingomonas sanxanigenens*	WP_025293719	52	5.00E-22
72	-	35,015	35,344	330	109	hypothetical protein	*Sphingomonas sanxanigenens*	WP_025293718	55	1.00E-08
73	-	35,316	35,633	318	105	hypothetical protein	None	n/a	n/a	n/a
74	-	35,630	36,505	876	291	endolysin	*Caulobacter* phage Sansa	AKU43454	50	2.00E-39
75	-	36,552	36,776	225	74	hypothetical protein	*Sphingomonas* sp. ATCC 31555	WP_019371220	68	2.00E-12
76	-	36,935	37,528	594	197	hypothetical protein	*Sphingomonas* sp. ATCC 31555	WP_019371221	44	2.00E-34
77	-	37,589	39,856	2268	755	D-alanyl-D-alanine carboxypeptidase	*Methyloceanibacter caenitepidi*	BAQ15659	33	2.00E-25
78	-	39,853	40,308	456	151	hypothetical protein	*Delftia* sp. RIT313	WP_052155377	54	2.00E-27
79	-	40,309	43,059	2751	916	virion structural protein	*Pseudomonas* phage PaMx28	ALH23633	44	0.00E + 00
80	-	43,088	43,282	195	64	tail assembly protein	*Burkholderia* phage AH2	YP_006561132	53	1.00E-15
81	-	43,279	43,509	231	76	virion structural protein	*Pseudomonas* phage PaMx25	ALH23804	77	5.00E-17
82	-	43,509	44,288	780	259	virion structural protein	*Pseudomonas* phage PaMx28	ALH23630	48	2.00E-72
83	-	44,285	45,874	1590	529	tail assembly structural protein	*Pseudomonas* phage MP1412	YP_006561079	35	1.00E-55
84	-	45,871	49,170	3300	1099	tail tape-measure protein	*Paracoccus* phage vB_PmaS_IMEP1	YP_009126438	51	3.00E-41
85	-	49,462	49,974	513	170	hypothetical protein	*Roseobacter* phage RDJL Phi 2	AKQ75858	26	8.00E-06
86	-	50,038	51,579	1542	513	major capsid protein	*Roseobacter* phage RDJL Phi 1	YP_004421846	46	3.00E-138
87	-	51,592	52,032	441	146	phage structural protein	*Roseobacter* phage RDJL Phi 1	YP_004421845	30	9.05E-13
88	-	52,029	52,526	498	165	virion structural protein	*Pseudomonas* phage PaMx25	ALH23810	34	6.00E-06
89	-	52,530	53,045	516	171	hypothetical protein	None	n/a	n/a	n/a
90	-	53,136	54,083	948	315	hypothetical protein	*Caulobacter* phage Sansa	AKU43432	61	9.00E-06
91	-	54,105	55,115	1011	336	major capsid protein E	*Pseudomonas* phage KPP23 (*Caulobacter* phage Sansa)	BAO53114 (AKU43431)	32 (24)	8.00E-39 (9.00E-12)
92	-	55,201	55,578	378	125	hypothetical protein	None	n/a	n/a	n/a
93	-	55,624	56,901	1278	425	hypothetical protein	*Roseobacter* phage RDJL Phi 2	AKQ75851	38	3.00E-21
94	-	56,891	57,370	480	159	hypothetical protein	*Roseobacter* phage RDJL Phi 2	AKQ75850	46	1.00E-23
95	-	57,494	58,489	996	331	head morphogenesis protein	*Roseobacter* phage RDJL Phi 2 (*Caulobacter* phage Sansa)	AKQ75849 (AKU43427)	49 (25)	4.00E-80 (7.00E-26)
96	-	58,494	58,892	399	132	hypothetical protein IB60_17100	*Brucella abortus* LMN1	KFH18426	31	5.00E-08
97	-	58,892	59,431	540	179	hypothetical protein (tail protein)	*Roseobacter* phage RDJL Phi 2 (*Caulobacter* phage Sansa)	AKQ75847 (AKU43445)	34 (57)	2.00E-09 (1.00E-03)
98	-	59,431	60,993	1563	520	portal protein	*Caulobacter* phage Sansa	AKU43426	25	5.00E-18
99	-	61,152	61,415	264	87	hypothetical protein	None	n/a	n/a	n/a
100	-	61,878	63,512	1635	544	terminase	*Agrobacterium rhizogenes*	WP_051696780	56	3.00E-148
101	-	63,493	64,023	531	176	hypothetical protein	*Nitratireductor basaltis*	WP_051913838	30	6.00E-07
102	+	64,145	64,327	183	60	hypothetical protein	None	n/a	n/a	n/a
103	+	64,371	64,556	186	61	hypothetical protein	None	n/a	n/a	n/a
104	+	64,553	64,687	135	44	hypothetical protein	None	n/a	n/a	n/a
105	+	64,747	65,037	291	96	hypothetical protein	None	n/a	n/a	n/a
106	+	65,037	65,204	168	55	hypothetical protein	None	n/a	n/a	n/a
107	+	65,233	65,520	288	95	hypothetical protein	None	n/a	n/a	n/a
108	+	65,532	65,675	144	47	hypothetical protein	None	n/a	n/a	n/a

Eight genes were found to encode proteins related to DNA metabolism. In addition to DNA modification methylase (ORF 1) and DNA methylase (ORF 67), phage vB_EliS-R6L encodes another three methylase proteins, including a methyltransferase (ORF 34), a type I restriction-modification (R-M) system methyltransferase subunit-like protein (ORF 40), and a cytosine-specific methyltransferase (ORF 55). The identities range from 31 to 56% (46% on average). Four of the five ORFs are predicted to contain a single domain, including a CcrM-like domain (ORF 1, with recognition site of GANTC), two SAM methyltransferase domains (COMT-like) (ORF 34 and 40, a versatile enzyme with various target molecules), and a Dam subfamily domain (ORF 67, with a recognition site of GATC). Methyltransferases are ubiquitous in prokaryotic genomes, and these enzymes are often associated with a cognate restriction endonuclease, forming an R-M system that protects bacterial cells from invasion by foreign DNA such as phages. Approximately 20% of annotated phage genomes encode methylases, and it is proposed that they may help the phage overcome R-M and other phage-targeted resistance systems in the host and prolong the effectiveness of infection [37]. As predicted by REBESE software, one R-M pair was recognized in the genome of *E. litoralis* DSM 8509 (with the recognition site CCGGAG), and five pairs were found for *E. longus* DSM 6997 (two of which have recognition sites GGCGCC and CGATCG; the other three have no recognition sites). Those R-M recognition sites indicate 37 potential cleavage sites (23 for CCGGAG, 14 for CGATCG) in the genome of phage vB_EliS-R6L. The predicted recognition site GATC of ORF 67 in the phage genome agrees with the R-M sites of DSM 6997, demonstrating the potential to overcome the host R-M system. Previous studies also found that phage T4 encodes a DAM methylase that targets GATC sites, protecting the phage DNA from an R-M system that recognizes this sequence [38]. Based on REBASE searches, 1051 homologs matched with the five methylase proteins, suggesting that R6L-like methylases are widespread, which may enhance infectivity and evasion of the host R-M system. Phage vB_EliS-R6L may represent a good model for exploitation of phage methylases and marine host-phage interactions. Moreover, Dziewit et al. (2014) suggested that methylases may account for differences in the methylation state and induce host transcriptional changes that are essential for the phage life cycle [39].

**Fig. 4:**
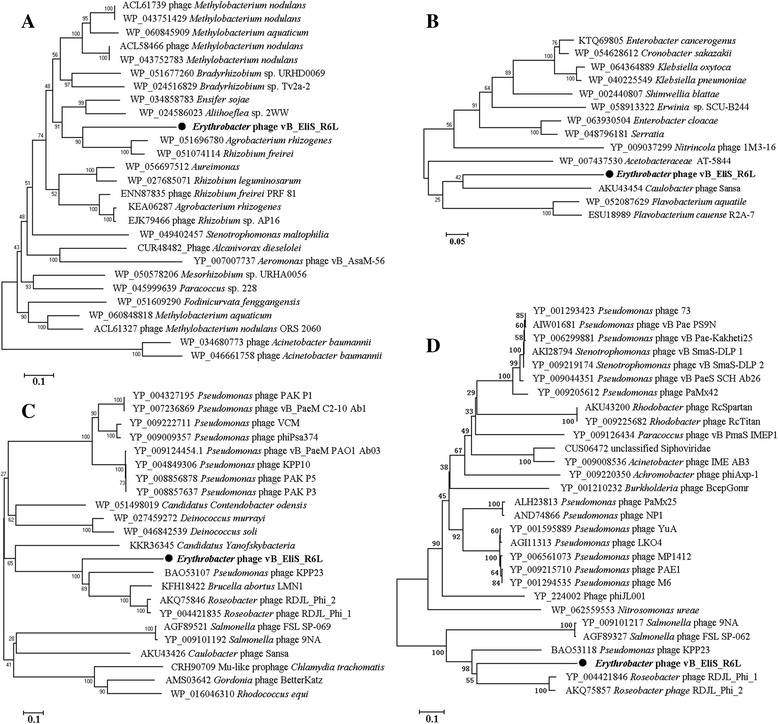
Phylogenetic relationships of four genes of vB_EliS-R6L-like phages. The neighbor-joining trees were based on the ClustalW alignment of amino acid sequences by MEGA 6.06. The bootstrap values were based on 200 replicates. (**a**) terminase; (**b**) endolysin; (**c**) portal protein; (**d**) major capsid protein

Twelve ORFs are predicted to encode proteins involved in the structure and assembly of virions, nine of which are homologous to genes from *Pseudomonas* (Gammaproteobacteria) and/or *Roseobacter* isometric siphophages [21, 40, 41]. A further four conjunctive ORFs with unknown functions also exhibit homology to these phage types. This is consistent with the results of the phylogenetic trees generated using major capsid protein and portal protein amino acid sequences (Fig. 4). However, it is noteworthy that except for these 13 ORFs, no other ORFs of vB_EliS-R6L show a high degree of homology to *Pseudomonas* or *Roseobacter* isometric phage sequences. It therefore appears that genes associated with the structural architecture of phage vB_EliS-R6L are relatively conserved and may have evolved independently from other genes in the genome. Moreover, the low protein identity predicted between phage vB_EliS-R6L and those homologies (26–77%, 41% on average), as well as clearly distant phylogenetic relationships (Fig. 4), suggest that phage vB_EliS-R6L exchanged genetic material with those closely related phages prior to a distinct evolutionary path.

One putative endolysin gene (ORF 74) and one molecular chaperone (ORF 9) were identified in the genome of vB_EliS-R6L, sharing 50% and 41% amino acid identity, respectively, with the corresponding proteins of the *Caulobacter* phage Sansa [42]. Most tailed phages achieve lysis via consecutive use two essential proteins, endolysin and holin (which control the length of the infective cycle). Endolysins are phage-encoded enzymes that degrade bacterial peptidoglycan. ORF 74 is predicted to contain one domain: a 176-aa region near the C-terminus that shows homology to proteins of the lysozyme-like superfamily. Although *Caulobacter* phage Sansa contains a lysis cassette (a holin/anti-holin pair and an endolysin) [42], none of the ORFs identified in phage vB_EliS-R6L exhibit homology to holin proteins. This may be the result of the limited number of holin protein sequences in databases [43, 44]. In addition, ORF 9 is predicted to contain one 49-aa domain homologous to chaperone J, which assists in translation.

Three ORFs are predicted to code for an acyl carrier protein (ORF 32), a nucleoside triphosphate hydrolase (ORF 41), and a phosphoadenosine phosphosulfate reductase (ORF 7). The acyl carrier protein in bacteria is responsible for fatty acid biosynthesis, requiring 4′-phosphopantetheine as a covalently attached cofactor. Acyl carrier protein homologs have also been identified in several other phages [45], though their function remains unclear. ORF 41 of phage vB_EliS-R6L is predicted to include a 292-aa P-loop domain of nucleoside triphosphate hydrolases, which hydrolyze the beta-gamma phosphate bond of a bound nucleoside triphosphate, providing energy for viral metabolism. ORF 7 shows 49% identity to phosphoadenosine phosphosulfate reductases, which have been identified in phages such as *Lactobacillus* phage AQ113 (GenBank accession no. HE956704) [46], *Mycobacterium* phage Baka (GenBank accession no. JF937090) [47], and *Pseudoalteromonas* phage PHS3 (GenBank accession no. KX912252, unpublished). Phosphoadenosine phosphosulfate reductases are thought to be involved in sulfate activation for cysteine biosynthesis. However, no studies have investigated the relationship between the activity of these enzymes and phage metabolism [46, 47].

Based on NCBI BLAST gene annotation results, phage vB_EliS-R6L shares 12 similar ORFs (E-value <10^–^^5^) with the *Caulobacter* phage Sansa, and another 5 pairs with an E-value <10^–^^3^ were found [42] (Table 2). The 12 homologous ORFs include 3 involved in DNA metabolism, 3 structural proteins, 1 methylase, 1 endolysin, 1 nucleoside triphosphate hydrolase, 1 molecular chaperone and 2 proteins of unknown function. However, the identities of the 12 pairs are not high (ranging from 23 to 61%; 34% on average), providing further evidence for the novelty of phage vB_EliS-R6L.

Endolysin protein (ORF 74), major capsid protein (ORF 91), portal protein (ORF 98), and terminase (ORF 100) were chosen for phylogenetic tree construction (Fig. 4). With the exception of the tree based on the terminase protein, in all cases, vB_EliS-R6L clusters with virulent bacteriophages, such as the *Caulobacter* phage Sansa, roseophages, and *Pseudomonas* phages. However, the clearly distant phylogenetic relationships with other phages suggest that vB_EliS-R6L is a novel phage. In the terminase-based tree, phage vB_EliS-R6L is located near prophages from *Agrobacterium rhizogenes* and *Rhizobium freirei*, agreeing with the BLASTp analysis. In addition, the phage life style predicted by the PHACTS algorithm indicated that it may be a temperate phage. However, no integrase, repressor, or other genes related to the SOS response [48] were identified in the genome of phage vB_EliS-R6L.

### Environmental distribution

**Fig. 5:**
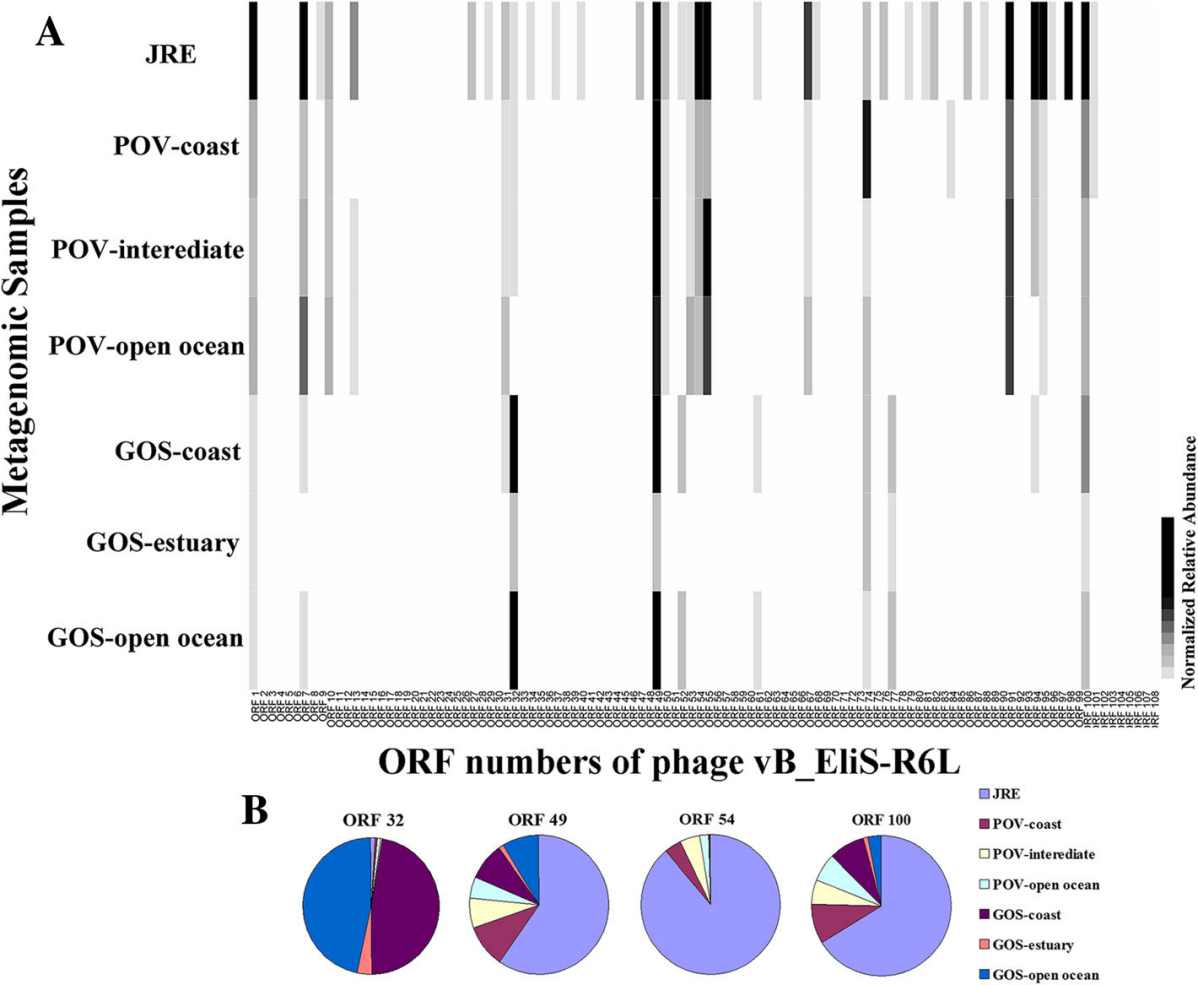
Relative abundance of vB_EliS-R6L -like phage genes in the metagenomes. (**a**) Heatmap of the normalized relative abundance of vB_EliS-R6L ORFs identified in the Jiulong River Estuary, Xiamen, China (JRE), Pacific Ocean Virome (POV) and Global Ocean Survey (GOS). (**b**) Normalized relative abundance of ORF 32, 49, 54 and 100 in the metagenomes

Metagenomic analysis indicated that vB_EliS-R6L-like phages are widespread in the examined environmental samples (Fig. 5). Across all metagenomic samples (JRE, POV and GOS), 7138 reads were successfully assigned and detected at rates of 10^–^^9^ to 10^–^^7^ per amino acid pair in the databases. The greatest matches were found in JRE (1.13 × 10^–^^7^ per pair), from which phage vB_EliS-R6L was isolated, followed by POV (1.94 × 10^–^^8^ per pair) and GOS (1.89 × 10^–^^8^ per pair) coastal samples. This is in agreement with the general distribution of *Erythrobacter* in the costal environment [1, 2, 49]. Forty-five ORFs were matched to homologs in the databases. The most relative abundant distribution was for ORF 49 (single-stranded DNA-binding protein, with function of DNA replication/repair, 5.28 × 10^–^^9^ per pair), ORF 54 (hypothetical protein, 3.05 × 10^–^^9^ per pair), ORF 32 (acyl carrier protein, 3.18 × 10^–^^9^ per pair) and ORF 100 (terminase, 5.07 × 10^–^^10^ per pair). Although the homologs of some ORFs (e.g., 9, 37, 45, 76, 81, 93 and 96) were only found in the JRE virome and/or the POV and GOS coastal samples, the hits for the most matched ORFs covered all three databases. This result suggests that vB_EliS-R6L is a previously unknown phage group that is widely distributed in the marine environment and that it could serve as a good reference for the taxonomic binning of marine viromes in the future.

## Conclusion

Phage vB_EliS-R6L is the first virus identified that can infect marine bacteria belonging to the genus *Erythrobacter*. The phage has a wide temperature and pH tolerance. With a 65.7-kb genome encoding 108 putative gene products, phage vB_EliS-R6L is novel among the cultured phage community and is largely different than all other known phages. Phage vB_EliS-R6L encodes five methylase proteins, suggesting the potential to overcome host resistance systems. Auxiliary metabolic genes in the phage genome were also annotated, such as those coding for an acyl carrier protein and phosphoadenosine phosphosulfate reductases. Metagenomic database queries suggest that vB_EliS-R6L-like phages are widely distributed in the marine environment, especially in coastal waters. *Erythrobacter* comprises one of the important clades of AAPBs [50, 51] and could represent the predominant AAPBs in the upper oceans [7]. Our study provides the basis for in-depth investigation of host-virus interactions and the ecological behavior of marine *Erythrobacter*.

## Abbreviations

AAPB: Aerobic anoxygenic phototrophic bacteria
SM: Sodium chloride-magnesium sulfate
TEM: Transmission electron microscopy
PFU: Plaque forming unit
EOP: Efficiency of plating
OD: Optical density
PCR: Polymerase chain reaction
ORFs: Open reading frames
R-M: Restriction-modification
JRE: Jiulong River Estuary
POC: Pacific Ocean Virome
GOS: Global Ocean Survey
